# The electrical characteristics and conduction mechanisms of Zn doped silicon-based Schottky barrier diode

**DOI:** 10.1016/j.heliyon.2023.e22793

**Published:** 2023-11-23

**Authors:** D.A. Oeba, J.O. Bodunrin, S.J. Moloi

**Affiliations:** a Department of Physics, Faculty of Science, Egerton University, P.O Box 536-20115, Egerton, Kenya; b Department of Physics, College of Science, Engineering, and Technology, University of South Africa, Private Bag X6, Florida, 1710, South Africa

**Keywords:** Silicon-based diode, Zn-doping, Conduction mechanism, Ohmic behaviour, Full depletion voltage

## Abstract

In this study, we investigated the effects of Zn doping on electrical properties and conduction mechanisms of *n*-silicon (*n*-Si) diodes using current-voltage (*I–V*) and capacitance-voltage-frequency (*C–V*–*f*) measurements. The results revealed that Zn doping alters the *I–V* behaviour of the diode from a typical exponential curve to an ohmic one. Notably, Zn doping increased the reverse current by a factor of 37, while reducing the forward current by a factor of 3 at 3 V. This suggested that Zn-related defects introduced more minority carriers into the Si. The introduction of minority carriers is confirmed by a change in material conductivity-type from *n*-to *p*-type. Moreover, Zn doping reduces the full depletion voltage (FDV), meaning the diode could be fully depleted with a lower voltage. This reduction in FDV was crucial for designing highly sensitive radiation detectors. The observed changes in the diode's electrical behaviour were attributed to defects introduced by Zn into Si. Zn-doped *n*-Si diodes exhibited characteristics akin to radiation-resistant diodes. These findings implied that Zn may be instrumental in advancing research focused on enhancing silicon properties and developing radiation-resistant detectors for high-energy physics studies.

## 1 Introduction

Various research works have sought to enhance the radiation tolerance of Si by incorporating costly dopants like platinum (Pt), gold (Au) [1,2], and lithium (Li) [3]. Although these metals have improved the material's radiation-hardness, detectors made from metal-doped Si suffer from high leakage current, resulting in increased electric noise, reduced detection sensitivity, and poor energy resolution [4,5]. As a result, researchers are now seeking alternative dopants in case Au and/or Pt are deemed too expensive or unavailable for research purposes. Identifying suitable dopants could lead to cost-effective and more reliable Si detectors for high-energy physics experiments [5].

To improve the efficiency of the radiation detector, Si has been doped with Zn of different concentrations [6]. Due to its lower ionization potential, Zn-doped detectors exhibit higher energy resolution due to a smaller detecting volume [6]. However, there is limited literature on the impact of Zn-doping on the electrical characteristics and conduction mechanisms of *n*-Si-based diodes [7,8], particularly on *I–V* and *C–V*–*f* properties.

Studies have shown that Zn diffuses into Si by exchanging interstitial and substitution atoms [9,10]. Zn introduces defect levels within the Si bandgap at $E_{V}$ at + 0.24 eV [8], $E_{V}$ + 0.55 eV and $E_{V}$ + 0.31 eV [11,12]. Additionally, doping Si with Zn generates acceptor levels at $E_{C}-$ 0.49, +0.60, and +0.33 eV [13]. Therefore, it is anticipated that Zn will increase/decrease the resistivity/conductivity of *n*-Si by generating defects that lead to the recombination of majority carriers. The Zn-induced defect level at 0.55 eV in the Si bandgap is close to mid-gap defect (0.56 eV), defects common to Pt and Au-doping found to be responsible for the improvement of the radiation-hardness of Si [1,2]. Hence, Schottky-based diodes (SBDs) fabricated on Zn-doped Si may exhibit properties comparable to Au- and Pt-doped Si, making Zn a viable and cost-effective dopant for scientific research. This mid-gap defect level interacts with the conduction and valence bands, resulting in an intrinsic-like material [14,15]. Detectors fabricated on intrinsic-like material are radiation-tolerant [15].

This study focused on fabricating SBDs on undoped and Zn-doped *n*-Si. The study investigated the effects of Zn doping on the electrical properties of SBDs. The effects of Zn-doping on diode parameters such as ideality factor (η), saturation current ($I_{s})$, series resistance ($R_{s})$, Schottky barrier height ($\Phi_{B}$), shunt resistance ($R_{\text{sh}})$ and doping concentration ($N_{D}$) were studied. The *C–V* measurements were taken at different frequencies to investigate the effect of frequency on the measured capacitance of the diodes.

## 2 Experimental details

### 2.1 Sample preparation

An *n*-Si wafer obtained from Semiconductor Wafer Inc., with a 275 ± 25.0 μm thickness was used in this work. The wafer was diced into smaller pieces measuring 6 mm × 6 mm. Before Zn doping, the pieces underwent a standard cleaning procedure [16]. The ion implantation for Zn doping was carried out using an ion implanter at iThemba LABS, Gauteng, South Africa. The implantation parameters were 160 keV and 1.0 × 10^17^ ion cm^−2^ for energy and fluence, respectively. The parameters were chosen to induce a significant number of defects deep in the Si material [17,18]. The expected range and maximum implantation depth were predicted using Stopping and Range of Ions in Matter 2013 [18] as 117.8 nm and 250 nm, respectively.

### 2.2 Diode fabrication

**Fig. 1:**
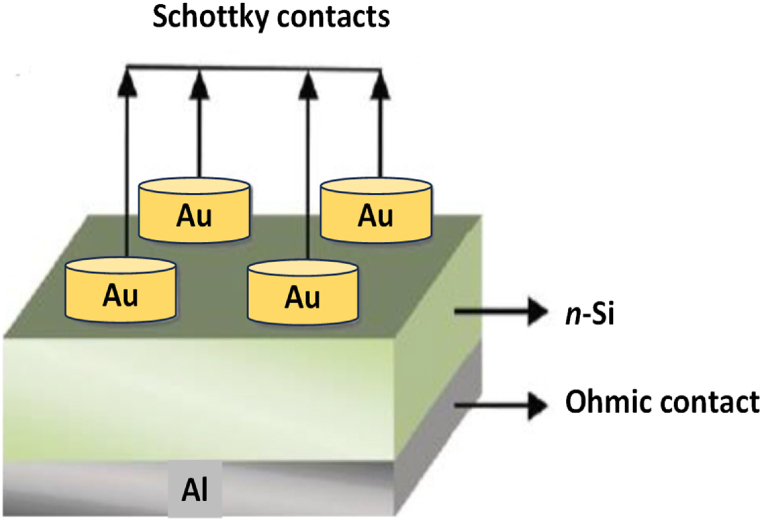
The schematic of fabricated diode with Au and Al as Schottky and ohmic contact, respectively*.*

Before forming the ohmic and Schottky contacts, the undoped and Zn doped *n*-Si samples underwent cleaning following standard procedures and were blow-dried using nitrogen gas. The formation of an ohmic contact was achieved by depositing a 100 nm thin layer of aluminium (Al) onto the rear surface of the wafers. The samples were annealed at 375 °C for 40 min in a vacuum to optimize the ohmic contact and reduce resistivity at the back contact. For the Schottky contact, a 130 nm thick layer of Au was deposited on the polished side of the *n*-Si material through a mask with 0.06 cm radius holes. The deposition occurred at a rate of 1 A s^−1^ under a 10^−6^ mbar pressure. The thermal vacuum deposition apparatus used to form the Schottky and ohmic contacts in this work is an Edwards AUTO 306 vacuum deposition and Fig. 1 depicts the schematic of the fabricated diode.

### 2.3 Diode characterization

*I–V* and *C–V* measurements were conducted at 300 K in dark conditions. The *I–V* measurements were taken over a voltage range of −3 V – 3 V to allow the dominance of tunnelling electrons over thermionic emission electrons [19]. The *C–V*–*f* measurements were recorded between 40 and 148 kHz [20]. This frequency range was chosen for this study after establishing that frequencies below 40 kHz or over 148 kHz led to unstable data. The measurements were performed at a fixed starting voltage of 0 V and a constant voltage step of 0.01 V across all samples to ensure consistency and uniformity during result analysis.

## 3 Results and discussion

### 3.1 Current-voltage

Considering the effects of the series resistance, $R_{s}$, the resulting current through the diode is given [7] as

(1) $$I=I_{S}\;\left[\exp\left(\frac{eV-IR_{s}}{\eta kT}\right)\right]\text{.}$$

The $I_{S}$ is determined by linearly fitting of ln(*I*)–*V* graph at zero bias and can be represented as:

(2) $$I_{S}=AA^{*}T^{2}\left(\frac{-e\Phi_{B}}{kT}\right)$$

where $A^{*}$ and *A* represents the Richardson constant and area of the diode, respectively. For *n*-Si, $A^{*}$ is 112 Acm^−2^ K^−2^ at room temperature.

To calculate the η, one can substitute the slope of the linear section of the forward ln(*I*)–*V* characteristic into the following equation:

(3) $$\eta =\frac{e}{kT}\frac{dV}{d\left(\ln\;I\right)}$$

where d*V*/d(ln *I*) stands for the reciprocal of the slope of the linear section in the ln(*I*)–*V* graph, the η is a measure of the ideal characteristics, with unity being the value for an ideal diode. However, in practice, η is greater than 1 [14]. Values of η above 1 indicate the involvement of other conduction mechanisms. The $\Phi_{B}$, can be determined using the evaluated value of $I_{S}$ from equation (2) as

(4) $$\Phi_{B}=\frac{kT}{e}\ln\left(\frac{AA^{*}T^{2}}{I_{S}}\right)\text{.}$$

The Cheung and Cheung approach was also used to determine η and $\Phi_{B}$ [21].

(5) $$\frac{dV}{d\left(\ln\;I\right)}=\left(\frac{\eta kT}{q}\right)+IR_{s}$$

(6) $$H\left(I\right)=V-\left(\frac{\eta kT}{q}\right)\ln\left(\frac{I}{{AA}^{*}T^{2}}\right)$$

and H(I) is given as

(7) $$H\left(I\right)=\eta \Phi_{B}+IR_{s}$$

**Fig. 2:**
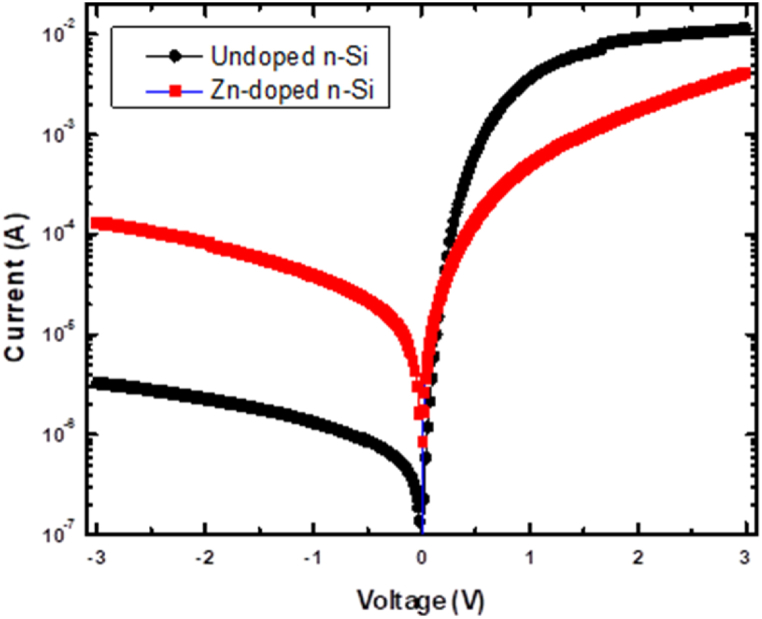
Semilogarithmic I–V behaviours of the fabricated SBDs.

Fig. 2 shows the semilogarithmic *I–V* behaviour of the fabricated SBDs. The undoped and Zn-doped diodes exhibit rectification behaviour, indicating successful fabrication. The reverse current shows a linear trend at low voltage regions and tends to saturate at higher voltages. This is expected since the reverse current is a result of minority carriers (holes) in *n*-Si. After Zn doping, the diode's reverse current increased by a factor of ∼37 at 3 V. A substantial increase in reverse current suggests that the defects due to Zn doping are responsible for the generation of minority carriers in *n*-Si. On the other hand, the forward current shows a linear increase with voltage but deviates from linearity at higher voltages for both diodes. The deviation is due to $R_{s}$ and the presence of interfacial layer between metal and Si [14]. The linear section is smaller in the Zn-doped diode compared to the undoped, suggesting that defects introduced by Zn in Si impact the diode's $R_{s}$. Fig. 2 shows that the forward current reduces by a factor of ∼3 at 3V after doping, indicating a recombination of majority carriers, leading to a drop in their concentration in the space charge region (SCR) due to Zn-induced defects.

**Fig. 3:**
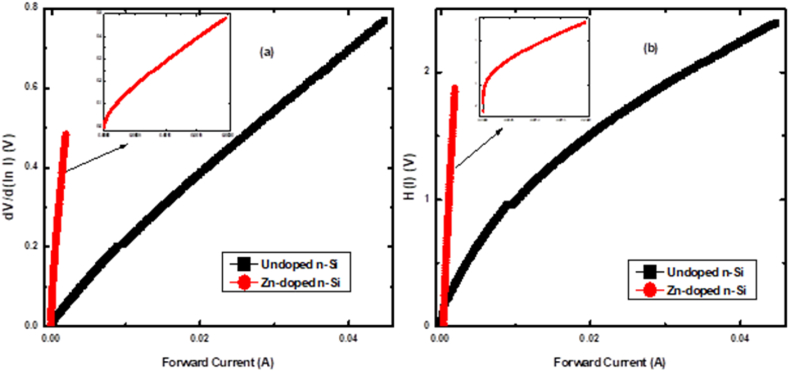
dV/d(ln I)– I (a), and H(I)–I (b) plot for the fabricated SBDs.

Fig. 3 (a and b) shows the $\frac{dV}{d\left(\ln\;I\right)}$ - *I* and H(I)-I plots of the undoped and Zn-doped *n*-Si SBDs, respectively. As per equations (5), (7), the graphs should be linear across the voltage regions, with $R_{s}$ representing the slopes. The values for η and $\Phi_{B}$ are evaluated from the y-axis intercepts of the H(I)-*I* and $\frac{dV}{d\left(\ln\;I\right)}$ – *I* graphs, respectively.

Parameters such as η, $\Phi_{B}$, $I_{s}$ and $R_{s}$ of the fabricated diodes were determined using ln(*I*)–*V* and Cheung's methods and are shown in Table 1. The parameters derived from the ln(*I*)–*V* graph were calculated from the linear region observed at low voltages, where the effect of $R_{s}$ is negligible. Conversely, the parameters obtained via Cheung's approach were calculated over the complete voltage region.

**Table 1:** The computed parameters of the Schottky barrier diodes.

ln (*I)–V*				$\frac{dV}{d\;\ln\left(I\right)}$*–I*		*H(I)–I*		*R*_i_*–V*		
SBDs	η	$\varphi_{B}$(eV)	$I_{s}$ (μA)	*R.R.*	η	$R_{s}$ (kΩ)	$\varphi_{B}$(eV)	$R_{s}$ (kΩ)	$R_{\text{sh}}$ (kΩ)	$R_{s}$ (kΩ)
**Undoped**	1.63	0.68	0.13	6870.00	1.54	16.17	0.71	38.78	926.42	0.28
**Zn-doped**	2.81	0.61	1.44	13.46	3.53	213.43	0.67	1103.5	26.59	0.70

**Fig. 4:**
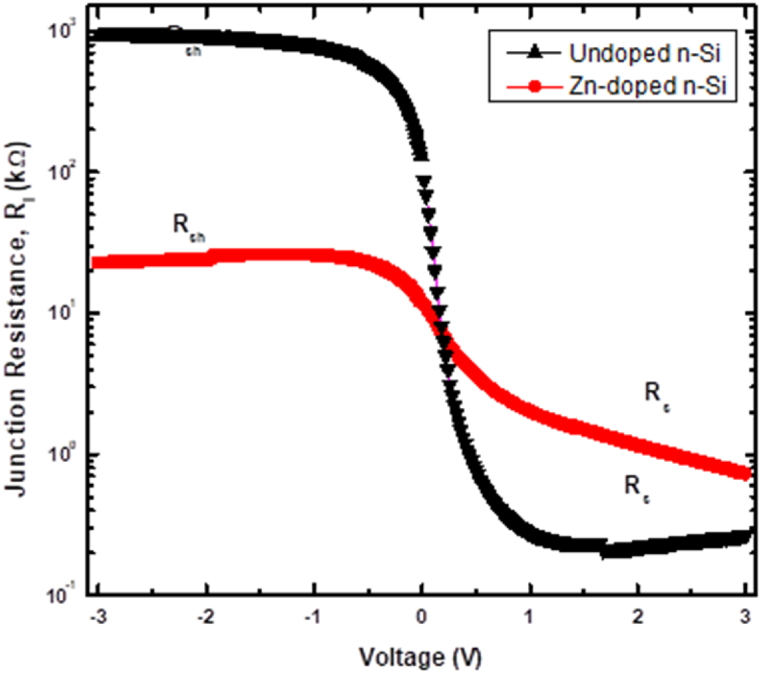
The diode resistance of the fabricated SBDs.

Fig. 4 shows the correlation between junction resistance, $R_{I}$ and voltage for the fabricated diodes. As can be seen in Fig. 4, $R_{I}$ stays steady as reverse voltage increases due to the extension of the SCR into the bulk. The $R_{I}$ for the doped diode is lower than the undoped diode because more charge carriers were injected into the SCR of the doped diode than the undoped diode. On the other hand, $R_{I}$ increases gradually with forward voltage for both diodes; this is attributed to the unimpeded carrier mobilities across the SCR. This effect is enhanced due to the decrease in the width of the SCR and the lowered $\Phi_{B}$ induced by forward biasing. For the doped diode, there is no noticeable saturation trend for $R_{i}$ in the forward bias, instead, it decreases gradually with voltage. This slight decrease implies a sluggish pace of charge carrier injection into the SCR, which may result from defects generated by the Zn doping in Si.

The η of undoped diode, evaluated from the ln(*I)–V* plot, is 1.63, which exceeds one, verifying the likely contribution of a tunnelling mechanism to charge distribution [22]. The η obtained from this work is higher than the 1.20 reported by Thebe et al. [23] for Pd/*n*-Si diodes and lower than the 1.79 reported by Bodunrin et al. [18] for Au/*n*-Si diodes. Additionally, the η of the undoped diode, evaluated using the $\frac{dV}{d\left(\ln\;I\right)}$ approach is 1.54, indicating the potential presence of other charge distribution mechanisms. In contrast, Güler et al. [22] reported a lower η of 1.34, while Thebe et al. [23] observed a slightly higher value of 1.55. These reported η values could be ascribed to the metal silicates or a possible unintentional SiO_2_ layer on the surface. These layers, potentially introduced by the metal-Si interface characteristics, may contribute to the higher η [14]. A minor variation of 0.09 in the η, obtained from both methods for the undoped diode, could be due to different regions used for parameter evaluation [18,23]. The η evaluated from ln(*I)–V* graph was evaluated at the low voltage regions, 0.05–0.5 V, where the influence of $R_{s}$ on the diode characteristics is minimal. On the other hand, the η evaluated using $\frac{dV}{d\left(\ln\;I\right)}$ approach was calculated on the entire voltage range. After Zn doping, the η calculated for the Zn-doped diode from the two methods (ln(*I)–V* and $\frac{dV}{d\;\ln\left(I\right)}$*-I*) is 2.81 and 3.53, respectively, have increased, indicating that other conduction mechanisms are involved in Si after doping with Zn.

The $\Phi_{B}$ of undoped diode was calculated using the ln(*I*)–*V* method and found to be 0.68 eV, which falls within the range reported in previous studies on Si-based diodes [[23], [24], [25], [26]]. Meanwhile, the $\Phi_{B}$ evaluated from the H(*I*)–*I* technique is 0.71 eV. In the case of the doped diode, both ln(*I*)–*V* and H(*I*)–*I* techniques yielded lower $\Phi_{B}$ values compared to the undoped diode. This observation agrees with similar trends reported by Msimanga and McPherson [15], where Au doping led to increased carrier density in the SCR. This increase in carrier density is due to minority carriers' generation in *n*-Si.After doping with Zn, the density of minority is so high, making the material's conductivity inverted from *n*-type to *p*-type. Regarding the $I_{S}$, the undoped diode exhibited a value of 0.13 μA, falling within an acceptable range [27,28] reported previously. On the other hand, the doped diode showed a higher $I_{S}$ of 1.44 μA, indicating that Zn induces defects that generates minority carriers in the bandgap of Si, leading to an increase in the saturation current.

The rectification ratio (R.R.) was used to establish how well the diodes were fabricated. The undoped diode demonstrated a high R.R., a value of 6870, consistent with previous report [29], indicating that our diodes are well fabricated. However, the R.R. for the Zn-doped diode reduced from 6870 to 13.46, likely due to the defects introduced by Zn in Si. The decrease in R.R. can be attributed to a decrease (increase) in the density of majority (minority) charge carriers in *n*-Si [18]. This variation of the density implies that in *n*-Si, electrons are compensated or recombined with Zn-introduced acceptor levels to decrease the forward current or minority carriers are generated to increase the reverse current.

The $R_{s}$ established from Cheungs and *R*_i_ *- V* plots showed discrepancies, which have been reported previously and attributed to an increase in the current generation rate at regions with high voltage [30,31]. This discrepancy suggests the presence of an insulating layer at the *m-s* interface. However, despite the differences in the $R_{s}$ values obtained from various methods, the value of $R_{s}$ increases after Zn doping. This increase is due to the defects introduced by Zn, which are responsible for charge carrier compensation and ultimately increase the resistivity of the Si material. The Schottky-based diodes' $R_{\text{sh}}$ and $R_{s}$ were determined from Fig. 4. In the reverse bias, $R_{\text{sh}}$ corresponds to the maximum $R_{i}$ value, whereas in the forward bias, $R_{s}$ corresponds to the minimum $R_{i}$ value. The computed $R_{\text{sh}}$ and $R_{s}$ values are provided in Table 1. The increased (reduced) values for $R_{\text{sh}}$ ($R_{s}$) on the undoped diode substantiate the diode's rectifying properties [32]. A decrease in $R_{\text{sh}}$ upon doping further demonstrates that doping *n*-Si with Zn results in the generation of holes.

**Fig. 5:**
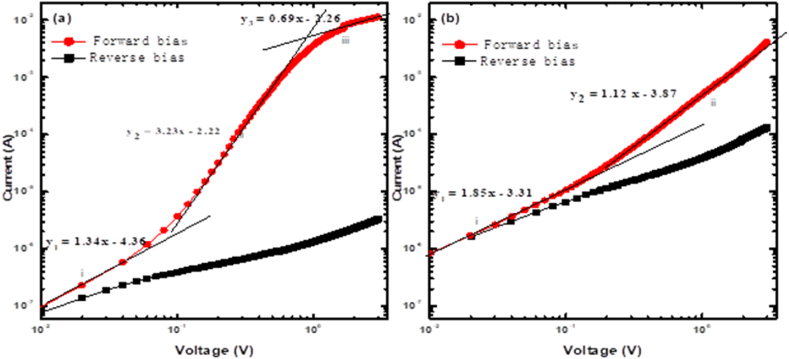
double logarithmic I–V plot of SBD fabricated on (a) undoped and (b) Zn-doped n-Si.

Fig. 5 shows a logarithmic *I–V* behaviour to study how the diodes' conduction mechanisms change as a result of Zn-doping. A gentle increase in reverse current is observed in Fig. 5, suggesting a slight dependence on the applied voltage. However, the forward current is higher than the reverse current, indicating that the charge carrier distribution within the material primarily results from recombination mechanisms [14,16,20]. A forward current trend for the undoped diode is shown in Fig. 5 (a) with three linear regions labelled (i), (ii), and (iii), each with a slope of 1.34, 3.23, and 0.69, respectively. Ohmic behaviour is indicated by a slope close to one, as seen in the first region [33]. A Trapped Charge-Limited Current (TCLC) with an exponential trap distribution is indicated by a slope more than 2, as seen in the region (ii) [33,34]. Lastly, the existence of $R_{s}$ at high voltages causes the slope in the third region to be lessened.

In the case of the doped diode presented in Fig. 5 (b), two regions (i and ii) were identified with their respective slopes of 1.85 and 1.12. The first region's slope is 1.85, approximating 2, which implies that this region is primarily influenced by the space charge limited current (SCLC). The slope of the second region has lessened. It approaches one, suggesting that this part is primarily governed by generation-recombination (*g-r*) centres, which leads to the diode displaying ohmic behaviour post-doping. Moreover, upon doping, the slope of the region (ii) has decreased from 3.23 to 1.12 at high voltages, owing to the creation of defect levels within the Si bandgap that function as recombination centres [7,20].

A noticeable reduction in the space between the trends of reverse and forward currents is seen in Fig. 5 (b), which indicates ohmic behaviour. Hence, the Zn-doped diode's ohmic behaviour indicates that introducing Zn in *n*-type Si also leads to defect formation in the middle of Si's bandgap (∼056 eV). This midgap defect level interacts with the conduction and valence bands, resulting in an intrinsic-like material [14,15]. Diodes exhibiting an intrinsic likeness resist radiation damage [14,15,17,24]. Therefore, apart from the defects responsible for minority carrier generation, Zn also introduces midgap defects in Si which is evident in the diode's ohmic behaviour.

### 3.2 Capacitance –voltage

The Capacitance (*C*) of the fabricated SBDs was evaluated at varying voltages (*V*) with different frequencies to investigate the effects of Zn-doping on the electrical properties of SBDs. The junction capacitance within these SBDs is given [22] as

(8) $$C=A\sqrt{\frac{e\varepsilon_{s}\varepsilon_{○}N_{D}}{2\left(V_{\text{bi}}+V\right)}}$$

where $\varepsilon_{s}$ is the semiconductor's dielectric constant, $\varepsilon_{○}$ is the dielectric constant of free space, $V_{\text{bi}}$ is the built-in-voltage, and $N_{D}$ is the doping density.

**Fig. 6:**
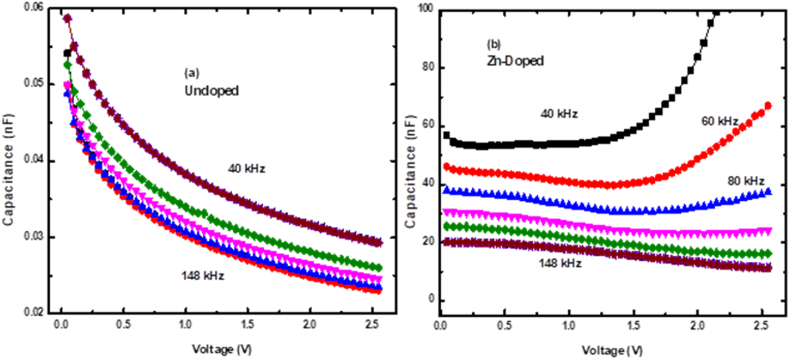
The C–V-f behaviour for (a) undoped and (b) Zn-doped n-Si SBDs.

The *C–V*–*f* behaviour of the fabricated SBDs across different measurement frequencies is shown in Fig. 6. Fig. 6 (a) demonstrates that the capacitance is relatively frequency-independent, which was anticipated given the material's lack of defects. Conversely, the capacitance of the doped diode is frequency-dependent due to the Zn-induced defects in the material [35]. As seen in Fig. 6 (a), there's a gentle decrease in capacitance at low voltage ranges, implying the charge carriers are being extracted from the SCR. There is no noticeable capacitance saturation for the undoped diode, suggesting that a 3 V isn't sufficient to deplete the SCR fully. Thus, a voltage above 3 V is necessary for complete SCR depletion. High FDV and the resulting electric field affects the speed at which the charge carriers drift in the detector, which can impact the noise characteristics and energy resolution of the detector [[35], [36]].

At low measurement frequencies, Fig. 6 (b) illustrates opposite trends to those of Fig. 6 (a), indicating that minority carriers predominantly influence the diode's conduction mechanism after Zn doping. This observation suggests that implanting Si with Zn changes the conductivity-type from *n*-to *p*-type by creating defects that induce minority carriers. A similar conductivity-type inversion has been documented in previous studies [37,38], demonstrating that Zn has similar effects in Si just like Pt and Au. The impact of these minority carriers is evident at low measurement frequencies after Zn doping. This change can be attributed to the relatively slow mobility of these carriers, which require more time (or lower frequencies) to align with the A.C. signal. However, at high-frequency measurements, the trend in Fig. 6 (b) are similar to that of Fig. 6 (a) due to the diminished impact of low-mobility carriers on the measured capacitance. Consequently, the measured capacitance primarily comes from majority carriers at higher frequencies due to their greater mobility than holes.

A comparison of trends between undoped and doped diodes in Fig. 6 reveals a clear difference. Unlike the undoped diode, which displays non-saturation tendency across all frequencies, the doped diode demonstrates a capacitance saturation within the 0–1.5 V range. This saturation at low voltages after doping indicates that Zn-related defects decrease FDV. A reduction in FDV implies that after doping, achieving complete depletion of SCR width can be accomplished with lower applied voltage.

**Fig. 7:**
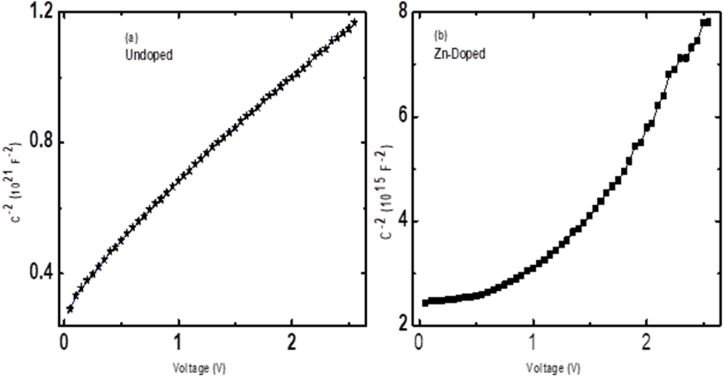
C ^−2^–V behaviour of (a) undoped and (b) Zn-doped n-Si SBDs*.*

The *C*^−2^–*V* behaviour of the fabricated SBDs presented in Fig. 7 is used to study the doping profiles at 148 kHz. A 148 kHz frequency was selected due to its minimal impact on interface charges influencing capacitance measurements [28,29]. We presented the distinction between linear regions in the *C*^−2^–*V* plots for undoped and doped diodes to examine the doping uniformity in the SCR. The *C*^−2^–*V* plot for undoped diode exhibited a linear region at voltages above approximately 0.25 V. In comparison, the plot for a doped diode had a linear region at voltages above about 1.4 V. A wide linear characteristic in the *C*^−2^–*V* plot of an undoped diode indicates that the doping density is uniform in the SCR [31]. The results obtained further provide insight into the quality of diode fabrication. The doping concentration for the undoped diode was found to be 4.46 × 10^15^ cm^−3^, resembling closely a previously reported value of 8.48 × 10^15^ cm^−3^ for a similar diode [28], further validating the quality of our diode.

**Fig. 8:**
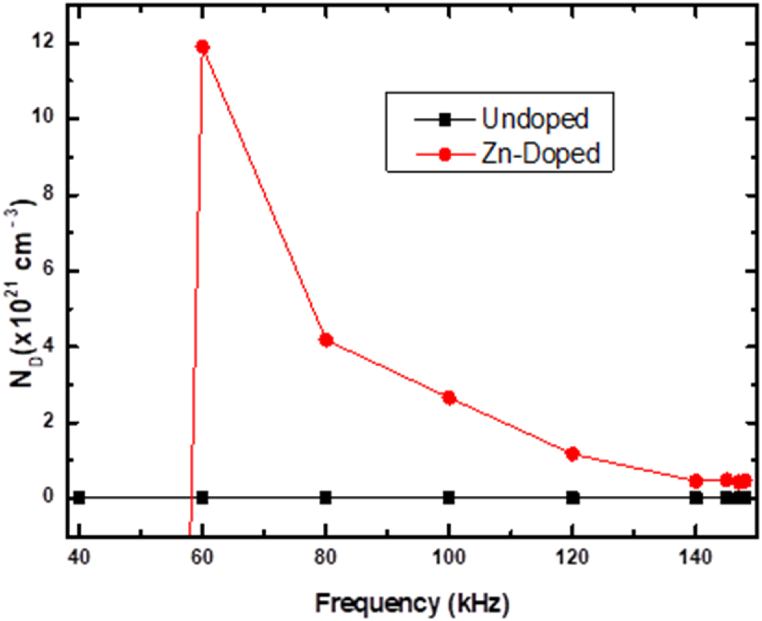
Variances in doping densities in response to the measurement frequency for the fabricated SBDs.

Fig. 8 illustrates the relationship between $N_{D}$ and frequency of the fabricated Schottky-based diodes. The data for the undoped diode demonstrates a minor variation as a function of measurement frequency, suggesting a lower defect density in the material that responds to frequency changes. For frequencies under 60 kHz, the Zn-doped diode shows a negative gradient, signifying a change in conductivity type. This change implies that additional minority carriers were introduced after doping Si with Zn. Importantly, no linear region was observed with a positive gradient for the Zn-doped diode at such low frequencies. As such, the diode's capacitance is primarily influenced by holes for the doped diode at 60 kHz. The Zn-doped diode trend also is above that of undoped, signifying an increase in the density of charge in the SCR following the doping process. As mentioned earlier, the increase can be attributed to the generation of holes in the material by Zn-induced defects.. Fig. 7 also displays a decrease in the $N_{D}$ of the doped diode as frequency increases, indicating that the lower mobility minority carriers become less active, and high mobility majority carriers majorly control the SCR capacitance as frequency increases.

## 4 Conclusion

In this study, undoped and Zn-doped *n*-Si diodes were successfully fabricated. Characteristics of the undoped diode were compared with similar diodes from other studies and found to fall within the range reported in previous studies on the same material. The *I–V* and *C–V*–*f* methods were utilized to examine the diode's electrical properties and the impacts of the Zn dopant in the Si material. These techniques provided complementary insights, revealing that defects induced by Zn lead to the generation of holes in Si. The generated holes compensate or recombine with the majority carriers, increasing material resistivity. The change in Si's conductivity type due to the generated holes is noticeable in low-frequency *C–V* measurements. Further, the study revealed that Zn doping reduces FDV, indicating that Zn doping might enhance the sensitivity of Si-based devices. The Zn-doped SBD exhibits characteristics analogous to devices found to be resistant to radiation damage, implying that Zn could effectively replace Au and Pt in enhancing Si properties. Replacing costly dopants with Zn would be beneficial in creating effective radiation sensors that meet current and future requirements.

## Data Availability

Data sharing is not applicable to this article as no new data were created or analyzed in this study.

## CRediT authorship contribution statement

**D.A. Oeba:** Formal analysis, Methodology, Investigation, Writing - original draft. **J.O. Bodunrin:** Formal analysis, Investigation, Writing - review & editing. **S.J. Moloi:** Conceptualization, Formal analysis, Funding acquisition, Methodology, Supervision, Writing - review & editing.

## Declaration of competing interest

The authors declare that they have no known competing financial interests or personal relationships that could have appeared to influence the work reported in this paper.
